# How to facilitate peer support – learnings from the development of a peer support program for people with T2DM via instant messaging service to improve diabetes self-management

**DOI:** 10.3389/fcdhc.2024.1491865

**Published:** 2025-01-06

**Authors:** Ursula Hemetek, Tatjana Aubram, Johanna Grüblbauer, Elisabeth Höld

**Affiliations:** ^1^ Institute of Health Sciences, Department of Health, University of Applied Sciences St. Pölten, St. Pölten, Austria; ^2^ Institute for Innovation Systems, Department Digital Business and Innovation, University of Applied Sciences St. Pölten, St. Pölten, Austria; ^3^ Institute for Creative\Media/Technologies, Department Media and Digital Technologies, University of Applied Sciences St. Pölten, St. Pölten, Austria

**Keywords:** diabetes self-management education and support, online peer support intervention, T2DM self-management, T2DM peer support, IMS communication strategy, instant messaging service health interventions

## Abstract

This study explores the use of Instant Messaging Services (IMS) for peer support among individuals with Type 2 Diabetes Mellitus (T2DM). Leveraging the popularity of IMS within the affected age group, the DiabPeerS study implemented a peer support intervention aimed at improving long-term blood glucose levels (HbA1c) in a randomized controlled trial (RCT). This article describes the development and acceptance of the IMS intervention used in the DiabPeerS study. The intervention included a communication strategy and content designed for lay moderators to facilitate group interaction among people with Type 2 Diabetes mellitus (PWT2D). The intervention’s acceptance was determined by conducting participant interviews, moderator meetings, and analysis of IMS chat protocols. Results indicate that the intervention was well-received, with participants engaging in meaningful exchange about diabetes self-management (DSM). However, those less familiar with online communication may benefit from preparational training and initial face-to-face meetings could enhance group cohesion. This research offers insights into the practical application of IMS for diabetes peer support, highlighting both its benefits and room for improvement.

## 1 Introduction

Diabetes mellitus is one of the four most important non-communicable diseases worldwide (1), and a drastic increase of its prevalence is anticipated especially for the most common Type 2 Diabetes mellitus (T2DM) (2). Patient self-management behaviors in particular play an important role in the treatment of the disease including everyday decisions on, e.g., eating habits, physical activity, medication adherence, stress control, and disease monitoring (3). Therefore, diabetes self-management education (DSME) and support (DSMS), defined as the ongoing process of “*facilitating the knowledge, skills, and ability necessary for diabetes self-care as well as activities that assist a person in implementing and sustaining the behaviors needed to manage his or her condition on an ongoing basis, beyond or outside of formal self-management training*” (4), play a significant role in increasing a patient’s self-management capacity and improving the effects of diabetes therapy (5–9). The terms DSM education and DSM support were combined to Diabetes Self-Management Education and Support (DSMES) to indicate the significant role of ongoing support (10). Even though DSME programs have been shown to improve clinical disease parameters like glycated hemoglobin (HbA1c) (11, 12) and even mortality (13), further research indicates that some of these outcomes might be difficult to maintain (14, 15) and seem to decline after the end of DSME programs (16). Consequently, effective strategies to preserve the effects of DSME are needed, and the provision of ongoing support for people with Type 2 Diabetes (PWT2Ds) shows a potential for this (4).

Peer support – offered by persons with experiential knowledge of the disease – offers an approach to provide cost-effective, low-threshold, and sustainable assistance for PWT2Ds (17). Peer support, within the health care context, is defined as: “*the provision of emotional, appraisal, and informational assistance by a created social network member who possesses experiential knowledge of a specific behaviour or stressor and similar characteristics as the target population, to address a health-related issue of a potentially or actually stressed focal person*.” (18p.329). Peer support for PWT2Ds is offered in various ways and has been proven to have positive effects on diabetes-related outcomes such as glycemic control, blood pressure, Body Mass Index (BMI)/body weight, physical activity (19, 20), or self-efficacy (21). The success of peer support appears to be due in part to the non-hierarchical, reciprocal relationship that is created through the sharing of similar life experiences (22), but even though there is a definition of peer support, and certain impact factors can be identified, there is no single form of implementation. Rather, peer support is offered in a wide variety of formats and settings: face to face, over the phone (e.g., text messages or telephone conversations), or online (e.g., email correspondence or apps) as well as in combination (17, 20, 22).

In recent years, online applications for T2DM peer support have been increasingly offered, researched, and proven to have positive effects on aspects of diabetes self-management (23–25). Compared to standard DSMES, web-based long-term support can be provided easily (26) and promptly (27), is inexpensive (26), and requires less effort to attend (28). Research on Diabetes Online Communities (DOC) shows that diabetes-related social networks can help patients to achieve positive behavioral changes by providing peer support among patients with similar conditions (29–31). In Austria, the age group most affected by T2DM onset (45-64 years) used Instant Messaging Services (IMS) on their mobile phones, while only 27.5% used other social media tools (32). Therefore, an IMS-based intervention operated on the mobile phone seems advantageous to reflect the customs of the target group (40+).

To our knowledge, most of the research dealing with diabetes-related peer support interventions via mobile phone focuses on so called mHealth or telehealth interventions, which usually labels interventions such as applications used as portable monitoring device, personal digital assistant as well as applications or web-based tools used to deliver educational materials (33–38). Typically, the latter applications for mobile or smartphone provide automated messages sent to the participant without allowing the receiver to start a live conversation (39). Thus, research on peer support interventions, which use mobile phone text messages as the primary mode of communication between patients and health care providers, indicates that interventions are more effective when direct interaction and/or regular feedback is provided (40, 41). IMS tools can provide this by using the conversation format for groups where participants have the opportunity to share their thoughts, needs, uncertainties, and experiences and provide feedback to each other, which – according to research on the definition of peer support (18, 22, 42) – is beneficial to facilitate peer support. Additionally, IMS facilitates connections between specified known users by adding them to specifically created groups that can only be entered upon invitation. Thereby, IMS technology offers invited users a so-called “safe space” to interact and engage in exchange with other peers. However, gaps in the exploration of “technology-mediated peer support, beyond voice-based telephones [and including] technology-mediated peer support modalities (i.e., video conferencing, SMS text message, social media)” have been identified (24), and research on how to facilitate peer support and/or DSMS via IMS is scarce.

In view of the described situation, the DiabPeerS Study (43) aimed to implement a peer-supported IMS RCT for patients with T2DM and to analyze the effects of an intervention on T2DM-related outcomes, primarily HbA1c (ClinicalTrials.gov Identifier: NCT04797429). Details on the RCT outcomes will be published elsewhere.

The aim of this article is to describe the development and implementation of the intervention (see chapter 2) in the DiabPeerS Study: The participants of the intervention group were part of a moderated IMS group and exchanged with other PWT2Ds on T2DM-related topics. For the IMS groups, technology from Mattermost (https://mattermost.com/) was used as it guarantees personal data protection, privacy, and the associated ethical principles. The IMS groups were moderated by a trained lay moderator equipped with a detailed manual and supervised by the study team.

Qualitative research results on how the intervention was perceived by moderators and participants as well as results on the operationalization of and reactions to the communication strategy via IMS from the analysis of chat protocols are provided in chapter 3 and discussed in chapter 4.

## 2 Materials and methods

### 2.1 Intervention development and theoretical underlining

For the intervention, a comprehensive and structured program in the form of a manual for the moderators with posting suggestions as well as training materials for moderators were developed by a research group including dietitians, nutritional scientists, diabetes advisers, communication scientists as well as a physician, a psychologist, and persons responsible for the diabetes-related disease management program “Therapie aktiv” in Lower Austria as reviewers.

#### 2.1.1 Developing a peer-led diabetes self-management support program for IMS

In a first step towards developing the intervention, a literature search was conducted on current recommendations for the theoretical basis and practical application of DSMES programs for PWT2D, taking into consideration that the intervention should provide DSM support rather than basic DSM education and should be seen as an addition to standard DSME programs. This research showed that patient empowerment and the promotion of self-management skills must form the basis for the conception of DSMES as a large part of therapeutic success lies in the hands of the patients (44). To facilitate this program, the content and communication strategy were fundamentally based on empowering and supporting participants in their leading self-management roles through various prompts in order to facilitate peer support and the exchange of experiences with other PWT2Ds, as well as enhancing competencies required for self-management and behavior change, such as making decisions, setting goals, planning appropriate actions, and addressing possible barriers through problem-solving (45).

To provide the appropriate structure and content for the program, the American National Standards for Diabetes Self-Management Education and Support (4) were followed. The DSMES Standards (4) suggest core content areas for DSMES programs which include reference to the ADCES7^®^ Self-Care Behaviors (46). While the ADCES7 ^®^ Self-Care Behaviors cover the topics of *Healthy Coping, Healthy Eating, Being Active, Taking Medication, Monitoring, Reducing Risk*, and *Problem Solving*, the DSMES Standards additionally recommend “*Diabetes pathophysiology and treatment options*” as a core topic, which was also included in the DiabPeerS intervention, to make sure that participants were on the same level of knowledge. The ADCES 7^®^ topic of “*Risk Reduction*” was covered within the topic of Self-Monitoring, while “*Problem-Solving*” was considered a Behavioral Change Technique (BCT) rather than a theme.

The application of Behavioral Change Techniques (BCTs) is also recommended for DSMES programs (4, 47). Thus, the application of BCTs was adopted in the DiabPeerS program to promote behavior change and to strengthen self-management competencies. Since the BCTs of Goal-Setting, Action-Planning and Problem-Solving are empathized in the DSMES standards (4) and manuals for training peer coaches (48, 49), they were adopted in the DiabPeerS manual: Once every month during the intervention, the moderators invited group members to set themselves their own SMART goal and plan an adequate action related to the actual theme. After four weeks, the goal was to be evaluated in the group, and potential problems were to be solved in a group problem-solving activity based on ADCES7^®^ (46).

Further research on the application of BCTs in T2DM online settings (50) showed that the BCTs of “Feedback on Performance”, “Providing Information on Consequences of Behavior”, “Barrier Identification/Problem-Solving”, and “Self-Monitoring” were the BCTs most often used and with the most promising outcomes, which also shaped the intervention design: “Feedback on Performance” was applied in the intervention as part of the communication strategy to promote user engagement by providing prompt and regular feedback (see below), while “Self-Monitoring” was integrated as a theme including aspects of “Providing Information on Consequences of Behavior” as well as “Risk Reduction” by providing content on necessary (preventive) check-ups and monitoring of feet, eyes, kidneys, etc.

**Table 1** shows the final program structure. For each of the seven core topics, online research was conducted by the study team to provide current – evidence-based on the Austrian Medical Diabetes Guidelines (7) – relevant, informative, and easy-to-understand content in the form of videos, articles, infographics, podcasts, etc. Eventually the content was compiled to a detailed pdf document, including a guideline and necessary instructions for the moderators.

**Table 1:** DiabPeers program structure.

Themes	Duration [weeks]
1. Getting to know each other and the program	2-3
Diabetes pathophysiology and treatment options	
2. T2DM pathophysiology, treatment and therapy	3
Lifestyle factors	
3. Physical activity	4-5
4. Healthy eating	8
Self-monitoring and check-ups in DSM	
5. Self-monitoring, secondary disorders, avoiding risks	3
6. Medication	1-2
Healthy coping	
7. Stress and psychological disorders	3
**Closing**	1
**Total**	**Approx. 28**

Explanation: For each topic a certain amount of time (in weeks) was calculated, and content was provided accordingly. However, moderators were instructed to handle this timeframe flexibly: if a topic met with little interest (measured by the response of the group members), the next one should be started and, equally, a topic could also be dealt with for longer if there was a lot of exchange on it. Group members and moderators were also encouraged to spontaneously contribute topics and questions that were of interest to them. In addition, questions e.g. regarding medication or nutrition, could also be asked if another group topic was being discussed.

#### 2.1.2 Communication strategy to facilitate user engagement for peer support on IMS

For the intervention, an IMS communication strategy had to be developed that facilitates peer support by engaging participants. Literature research on peer support was combined with an empirical approach to observe and adopt engaging communication strategies in Diabetes Online Communities (DOCs) on the social media platform Twitter (now X).

#### 2.1.2.1 Motives of people with diabetes to engage in DOCs – different types of peer support

Results of the literature review on the motives of PWDs (including people with type 1 and type 2 diabetes) to visit DOCs largely agree with the definition of peer support (the provision of “emotional, appraisal, and informational assistance”) provided by Dennis et al. (18). Thus, according to Oser et al.: “*The most common activities observed in DOCs are giving and receiving various types of support: psychosocial, technical, informational, and self-management.*” (31). Gavrila et al. (51) report similar observations on emotional support, technical support, and medical support, while Litchman et al. (24) identify six motivations for older adults visit DOCs: 1) information to improve self-care, 2) emotional support, 3) belonging to a community, 4) validation of information, 5) cause for concern, and 6) interaction with health care professionals.

To facilitate user engagement/motivation in the DiabPeers intervention, these motives were summarized into four types of peer support that should be provided/facilitated:

Emotional support is identified as the most important function of Diabetes Online Communities (DOCs) and is described in literature (31, 51, 52) as seeking mutual reinforcement to receive emotional support in difficult situations and feelings of sadness or frustration related to the disease. Additionally, the desire to provide this support to others is a motivation for many to participate in such groups. In DOCs, PWDs find a community of fellow patients who, unlike friends, family, and health professionals, understand first hand the range of issues surrounding the condition and empathize with all the emotions that arise from it.

Informational support, i.e., being able to request and receive information, is another benefit that participants in DOCs articulate and value. This does not only concern factual knowledge about coping with the disease, but, beyond that, the sharing of experiential knowledge: Other affected individuals can offer advice based on their “lived experiences” as a PWD (31). Furthermore, members of DOCs appreciate that they can validate information found elsewhere in this way: The diversity of first-hand accounts brings a variety of perspectives to a topic, thus enhancing one’s own knowledge about diabetes. Many feel that discussions within the community are more profound than what could be gleaned from information on the media (31, 52).

The quest for technical support is mentioned in the study by Gavrila et al. (51) and refers to dealing with various tools and instruments that are necessary for managing or facilitating the disease. Examples include handling various types of glucose meters or self-monitoring apps. In cases of uncertainty in handling these tools, group exchange of experiences and concrete, detailed guidance can be helpful.

Medical support naturally arises as a theme in DOCs, such as discussions about physical complaints related to diabetes or side effects of diabetes medication. Distinguishing between medical diagnosis and medication or therapy recommendations is not always straightforward. Additionally, there is a risk of providing tips that are generally harmful or harmful for specific individuals. While this problem did not occur in the studies of Litchman et al. (52) and Gavrila et al. (51), Grunberg (53) describes the need for peer facilitators to have strategies for handling certain posts appropriately and suggest regular monitoring by the study team as well as prompt consultation in cases of insecurity.

In the DiabPeerS intervention, the facilitation of emotional and informational support was promoted primarily through the selection of suitable content, engaging post formulation, and the creation of corresponding online materials (see 2.2.2.2). Technical support was encouraged by including subtopics on glucose meters and blood pressure monitors. Regarding medical support, moderators were instructed not to provide medical advice as a general guideline and to consult the study team if confronted with medical inquiries.

##### 2.1.2.2 Online health communication strategies to facilitate user engagement

Understanding the types of support sought and provided in T2DM online interventions and DOCs enabled a nuanced approach to selecting appropriate content for the manual. Further information on how to communicate and craft engaging posts in online or IMS interventions in order to promote interaction and engagement necessary to facilitate peer support was found in research on how to strategically engage users in health communication, with “Call-to-Action” (54) and “Nudging” (55) approaches. The following elements were incorporated into the manual, and moderators were instructed to:

- expect error (55) and therefore share helpful materials and checklists, e.g., for necessary regular medical check-ups, that were provided in the manual.
- set reminders (56), especially when it came to goal-setting activities, which were also indicated in the manual as part of the program.
- provide prompt and regular reactions and *feedback* to engage users and create a community atmosphere (51), and always try to formulate posts, reactions, and feedback in a positive, reinforcing, and motivating way (55).
- provide elements of *gamification* (57) like quizzes, common activities, and small challenges that were also provided in the manual as links to online quizzes or suggestions for common activities such as: “*Share your perfect healthy plate with the group*”.
- actively share personal experiences on, e.g., successful coping strategies to facilitate *social modeling*. Social modeling is based on the social learning theory which proposes that new behaviors can be acquired by observing and imitating others (58). To share *Personal Stories* is also suggested by Pedersen et al. (54) as part of the “Call-to-Action” communication strategy. Moderators were instructed to share their personal experiences and encourage group members to do the same.
- give participants the chance to *co-create* content (54) by, e.g., inviting them to complete shared sentences with their own words: “The last thing I did to feel good about myself was: (…)”.
- create “*engaging fact posts*” (54) by combining informative posts with activities like questions to stimulate discussion and exchange: “This article provides information on the positive effects of oats on blood sugar (….). What are your favorite recipes with oats?” In the manual, most posts provided were a combination of informative and interactive elements.
- use a “*welcoming tone of voice*” and avoid “*interrupting good conversations*” of participants (54), which was introduced to the moderators in training and in the manual.
- “*react promptly”* – suggested by Pedersen et al. (2020) – to critical posts to avoid the escalation of potential conflict and to avoid misleading information from spreading. In order to handle critical posts, it can also be helpful to “*Refer to house rules when users act disrespectfully*” (54), which was adapted for the moderators in the DiabPeerS program: “*Group rules*” concerning the avoidance of sharing fake news, commercial advertisements, to communicate in a respectful way and to avoid racist, sexist, homophobic, etc. comments and to refrain from sharing group content with others, were formulated and shared by moderators at the start of the intervention. All group members were asked to agree to those rules and were also invited to add rules, if needed.

**Figure 1:**
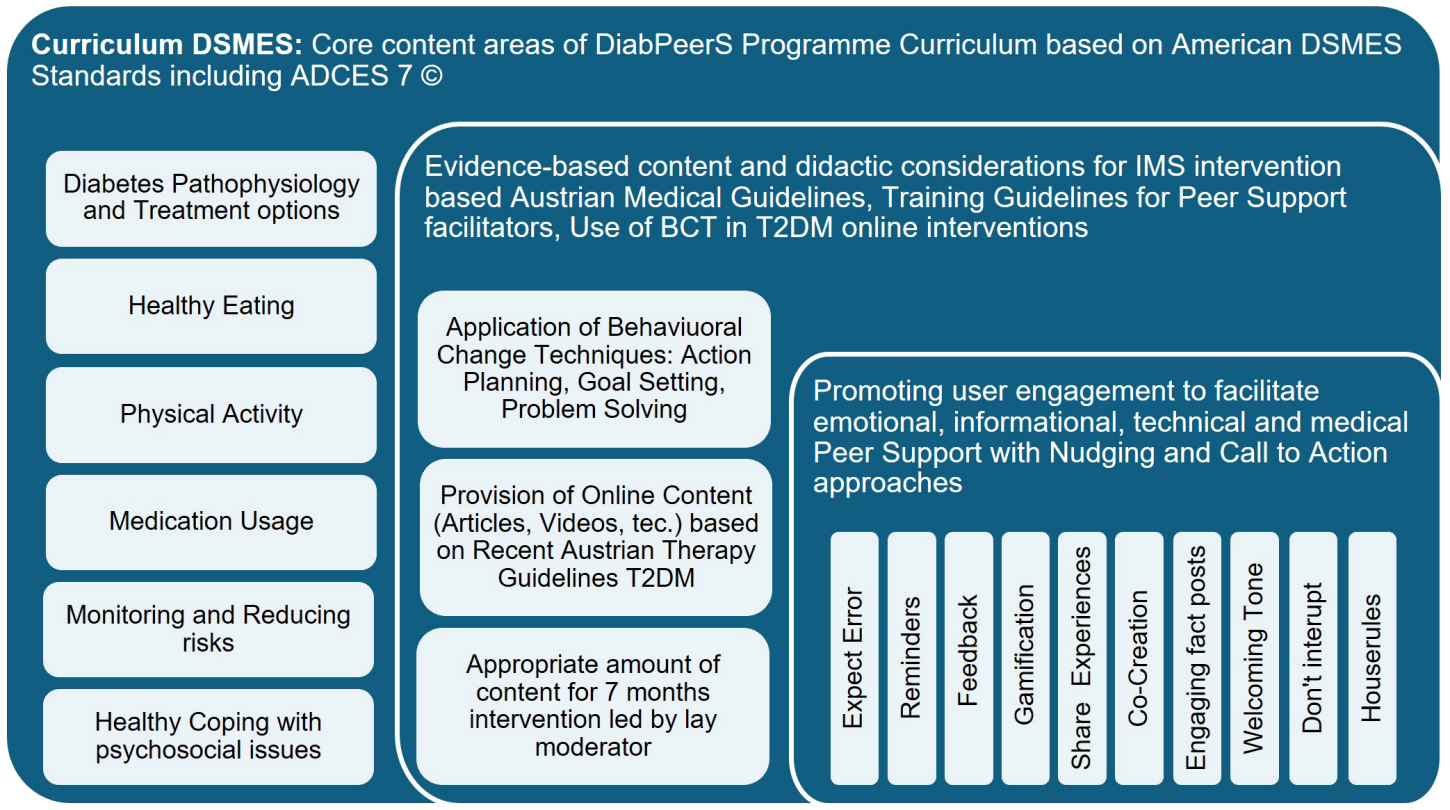
Visualization of the Development of the DiabPeerS Intervention Program.

Additional empirical research was conducted in DOCs pertaining to popular influencers (e.g., https://twitter.com/Heather_RoseW or https://mobile.twitter.com/wocdiabetes) or websites (e.g., https://beyondtype2.org) on the social media platform Twitter (now X) to observe best practice examples for Nudging, Call to Action, or Engagement. Posts that were identified as stimulating were selected, translated into German, and graphically edited to create sharable images for the manual. **Figure 1** visualizes the development process fo teh DiabPeerS programme.

### 2.2 Recruiting and training of moderators

Research on structural aspects of T2DM-related peer support (17) indicates that peer support interventions are usually guided/facilitated by persons who are trained to varying degrees, not health professionals but diagnosed with T2DM themselves, and who offer support on a voluntary basis, i.e., who are formally recognized but not monetarily compensated. In most cases, the qualification criteria for these individuals include an acceptable HbA1c – as an indicator of successful DSM – and, generally, the motivation to voluntarily support others in their DSM. Similar variability as in the implementation of peer support can also be found in the designation (peer supporter, peer educator, peer leader, peer support facilitator, etc.), training (between 2 and 46 hours), and roles of these individuals.

Further research was conducted on existing training materials for lay peer facilitators in a T2DM context available online and in the English or German languages. Training programs were found for diabetes peer supporters (48) or peer leaders (49), which focused on face-to-face settings. No training materials or research on detailed training content were available for online or IMS-based T2DM peer-led interventions, thus the programs were used for orientation purposes.

Besides structural aspects, research on role definitions of T2DM-related peer support facilitators (42) helped to sharpen the role description of the peer facilitators: The design of the DiabPeerS intervention should set the framework conditions to enable an exchange of experiences among those affected without reducing their social relationship to so-called “illness companions”, which would entail the risk of exchanging medically questionable and confounding subjective views (42). At the same time, peer support should not be equated with a classic patient-health professional relationship or training, in which content is conveyed by professionals in a directive manner, nor should peer facilitators slip into the role of “para-professionals” who act as extensions of the existing medical staff (42).

For the DiabPeerS intervention, it was intended to provide peer support allowing sufficient exchange of experience and depth of relationship but at the same time being directive to the extent that no incorrect content is conveyed. The task was to share predetermined content from the intervention manual combined with personal experiences and perspectives, to make it more tangible and authentic, to actively invite participants to take part in the group, to encourage group interaction and support, to pay attention to group dynamics, and to promote a trusting and respectful environment. It was not intended for the moderators to provide individual advice to participants.

The term “moderator” was used for the peer facilitators, as it is easy to understand in German and aptly describes the task. Recruitment criteria can be found in (43). Additional to the formal criteria potential moderators were interviewed by the study team with regard to: Personal interest, therapy adherence, commitment in moderating, using IMS and willingness to participate in 6-10 hours of training as well as willingness to cooperate with the study team. Many suitable candidates had professional or volunteer experience in management, coaching, or community work.

Three moderators were recruited, leading three IMS groups. The training covered approximately 10 hours divided into 3 training days over a period of two to three weeks and was conducted by a dietitian, a diabetes adviser, and a communication scientist at the St. Pölten UAS. In the training sessions, the moderators were encouraged to actively participate in discussions and practical exercises using teach-back methods – i.e. letting the learner explain or “teach-back” the newly acquired knowledge - and sessions spaced to allow practice with the IMS tool and manual. Topics included patient empowerment, self-management through peer support, skills development using BCT, and communication strategies for group dynamics, online interaction and digital media literacy.

### 2.3 Methods of data collection on intervention acceptance and user behavior

In this methodological section, information on data collection and analysis during and after the intervention is described, focusing on how the intervention program to facilitate peer support via an IMS application was perceived by moderators and participants. Several qualitative research approaches were used to this end.

#### 2.3.1 Supervision meeting with moderators

Supervision meetings with moderators were conducted by two members of the study team, who were responsible for the intervention development, moderators training and conduction of the measuring appointments and who constantly observed the IMS activities. Notes of these observations were regularly discussed by the two team members and integrated into the protocols, in preparation for the supervision meetings. The supervision meetings were conducted via MS Teams, lasted for about 30-60 minutes each, and were recorded in writing by one of the study team members following a semi-structured template, including the following topics: description/experience of the moderation task (including posting behavior), role understanding, perception of group interaction and peer support (engagement/motivation/dynamics). In total, minutes were kept for 13 meetings, and the results were summarized both inductively and deductively based on the topics of task management (use of manual and working structure), role understanding, and engaging participants.

#### 2.3.2 Interviews with participants of the intervention group

37 participants included in the intervention group and allocated into three IMS groups. For the duration of DiabPeerS Study (14 months including 7 months of IMS intervention) participants had to attend four measuring appointments. During the first measuring appointment, participants of the intervention group were introduced to the Mattermost app by a member of the study team, who installed the app on their mobile phone and explained its use. After the first three months of the intervention, all participants of the intervention group who attended the second measuring appointment (n = 24; T1 = 3 months after the start of the intervention. Due to illness or termination of study participation not all previously included participants of the intervention group (n=37) attended the second measuring appointment) were briefly interviewed about their experience in the group so far. In order to complement these broad range of perspectives with deeper insights on the perception of the intervention after completion of the intervention additional in-depth interviews were conducted (n=8; after T3 = 7 months after start of intervention).

##### 2.3.2.1 Brief interviews

The brief interviews were not taped but recorded in writing by the interviewers. The interviews were conducted as one part of the measuring appointments and prepared by one member of the study team (involved in development, observation of IMS-chatgroups and supervision meetings) but also two additional members of the study team conducted and protocolled interviews. Questions were structured as follows: experience of group and group interaction, motivation to post/participate, and obstacles to posting/participating. Following the method of structuring qualitative content analysis (59) the organizing researcher primarily coded interview protocols deductively – based on the interview questions - using the MAXQDA software (24 Release 24.1.0). Results were structured into three main themes: 1) motivation to post (both actively and passively), 2) perceived benefits, and 3) “What was missing?”. In correspondence with the research interest on the significance and perception of peer support in an IMS intervention and in thorough discussion with the study team, results on the themes were evaluated according to which type or element of peer support (informational, emotional, technical, medical, or sharing of experiences) they reflect. In a second step, based on how participants described their user behavior and their perception of the intervention roughly four different user types (active users, passive users, observers, and disappointed/rejecting participants) were identified (see **Table 2**).

**Table 2:** Typology of user types in DiabPeerS intervention at T1 (3 months after intervention start.).

Rejected/disappointed (n=5):	Do not post at all and stop reading after a short time. Reasons: rejection of online/phone-based communication, content not interesting/appealing (different expectations), technical difficulties
Observers (n=2):	Never/rarely post and do not respond to requests but are interested in the content and observation. Find useful/interesting content for themselves
Passive users (n=7):	Read along, do not post actively but primarily in response to prompts (from the moderator) and thus contribute to the group exchange. Find useful/interesting content for themselves
Active users (n=10):	Participate actively by contributing their experience/knowledge to the group, also ask questions and appreciate the exchange with other people concerned. They want to support others, but also find support in the group and useful/interesting content for themselves

##### 2.3.2.2 In-depth interviews

The rough typology of user types (**Table 2**) was the basis for the subsequent sampling process, aiming to select participants as interview partners, with maximum diversity concerning their posting behavior and perception of the intervention (after completion). Apart from posting behavior and perception of the intervention, the parameters of group allocation and sex were applied to the sampling of interview partners. Selected participants of the intervention group were contacted via telephone after the measurements T2/T3 and asked whether they would be available for an interview. All persons asked for an interview, agreed. In total, 8 interviews were conducted in July 2023. **Table 3** displays the final sample of interview partners including a corresponding representative statement on their experience of the intervention and a general indication on whether the intervention was perceived as beneficial or not.

**Table 3:** Description of interview partners for in-depth interviews after completion of DiabPeerS intervention.

Group	Posting behaviour	Perceived benefit
Group 2/IP28	Constant active and passive participation: “*I think it’s a really great thing. The exchange was excellent.”* (IP28, Pos. 9)	+
Group2/IP127	Rejecting – specific interest not fulfilled: “*I would like to say that I didn’t participate much in this group. I read it sometimes, I put a question in once that wasn’t actually answered.”* (IP127, Pos. 4)	–
Group3/IP15	Rejecting: Does not like or appreciate mobile phone texting and online communication (protocoled telephone interview)	–
Group3/IP101	Constant passive and occasionally active participation: “*Yes, it was fine. Well, I have to say that the moderator did a great job (…) tried very hard and provided us with very good information. In general, I found it very motivating to continuously receive reports.”* (IP101, Pos. 15)	+
Group3/IP46	Constant passive participation: “*I'm one of those people who say they'll join in. But I prefer to stand at the back and join in (…) The whole thing should flow along nicely.*” (IP46, Pos. 47)	+
Group1/IP14	Initially active, but then only passive participation (because of disappointment with the group interaction): “*So, if I had a topic, there was no response or no response at all and then of course I gave up at some point. I would say that I only responded towards the end.”* (IP14, Pos. 73)	–
Group1/IP16	Initially active participation, then reading along and disappointed/rejecting (with the content): “*Yes, I've tried it once or twice, but nothing really came back. For a while it was the photos of who was where. I'm less interested in that. (…) I had technical difficulties a few times, then I read along less. Then it worked again. But I did not post anymore.”* (IP16, pos.11 and pos. 25)	–
Group 1/IP144	Constant passive, occasionally active participation: “*My group was great. We were always kept informed by (…) The participation of the group was somewhat poor. I think there were only three of us (…) who answered and wrote regularly.”* (IP144, pos. 14)	+

All interviews were planned and conducted by one team member of the study team, strongly involved in intervention development, moderators training and supervision meetings, measuring appointments and brief interviews. Seven interviews were conducted via MS Teams, recorded, and transcribed. Due to technical difficulties, one interview had to be conducted via telephone and could only be recorded in writing. The semi-structured interview guide was comprised of similar but more in-depth questions about the personal experience of the group using a storytelling approach (60): “*How did you experience your participation in the DiabPeerS Study/IMS group? Please start from the beginning.*” in combination with more structured questions about topics of interest and/or posting motivation as well as ideas on how to improve the intervention: “*In a perfect world, what would the intervention look like?”*.

Case descriptions were created based on the interview transcripts, focusing on the overall experience/perception (perceived benefit) of the intervention. To build the coding frame, transcripts were loaded in the MAXQDA software (24 Release 24.1.0) and coded by two researchers of the study team, one who conducted the interviews and one who transcribed the interviews.

While one researcher coded the interview transcripts openly in MAXQDA, by selecting paragraphs or sentences of the transcripts with the mouse and assigning a code to it, the second researcher paraphrased relevant text passages by summarizing them to their meaning with a similar function in the program. In the following step paraphrases were coded and compared to the codes of the open coding process. Eventually the codes were defined and bundled and structured into themes, codes and subcodes. Direct anchor quotes of the interview partners (IPs) were identified as representative examples that illustrate core concepts for codes and categories during data analysis. All anchor quotes were translated from German into English by the authors (see **Table 4** Coding Frame).

**Table 4:** Coding frame of in-depth interviews.

Themes/ Categories	Codes	Subcodes
Moderation style/competence	Personality	Feeling of sympathy towards moderator important
	efforts/endeavours	Perceived effort leads to positive perception
		Perceived lack of effort leads to negative assessment
	Sensitivity for group dynamics	Need to facilitate and sustain group conversation
		Need to motivate group members
		(Prompt) reaction to posts of group members
	Neutrality towards shared content and in relation to group members	Neutrality perceived positive, if content is shared with neutrality and versatility
		Sharing information without personal comment can be perceived as negligent or uninvolved
		Irritation about perceived inequality in interaction with group members
IMS Setting	Incomplete/Declining participation barrier to Peer Support disappointing	Decline of participation after initial phase disappointing
		Perceived need to connect with other PWDs – build community
		Less participants- less exchange of experiences
	Anonymity of Group members	Barrier to active posting: Difficult to assess how posts are perceived by others-difficult to assess posts of others
		Advantage of anonymity: less inhibitions to post certain things
	Face-to-Face Meetings	Desired to counteract anonymity
		Desired for joint physical activity
	Flexibility (time/place)	in participation advantage (participation in own pace) and barrier (less engagement/commitment)
	Differences in Online/Phone Communication/Posting Behaviour	Partly no interest in Mobile Phone/Online Communication
		IMS perceived more effective and tailored to individual needs than Social Media
Peer Support	Desire to connect with other PWDs	
	Desire to exchange experiences with other PWDs	
Components of Peer Support	Emotional Peer Support controversies	More emotional support desired (not provided)
		Emotional Support perceived as unnecessary (other interest)
	Informative Support	High amount and variation of information appreciated
		Content perceived as “learning material”
		Information needs to be connected with experiential knowledge
		Patronizing and repetitive: “things you always get told”
	Medical Support	Delicate topic because of lay moderator
		Interest in very specific medical aspects and recommendations
		Unmet limited interest in medical aspects leads to reduced participation
		No exchange about GLP1 analogs
	Behaviour Change	adopting and successfully applying recommendations from other group members
		ideas require self-planned implementation for behaviour change

#### 2.3.3 Chat protocols of the IMS groups

To evaluate the effectiveness of the developed communication strategy, a content analysis of the IMS group’s chat protocols was conducted. Chat logs were exported as Excel files which were used for conducting the content analysis.

For the analysis categories were developed deductively based on research questions on the effectiveness of the communication strategy of the intervention. For this, all posts in the chat logs were coded focusing on different types of peer support (informational, emotional, technical and medical), topics of the posts (as outlined in the DiabPeers program), sharing of experiences, and (the initiation of) behavior change. Additional subcategories were developed inductively, depending on the content. Prior to the analysis, two coders conducted a pre-test with the first 10% of posts in the first IMS group. One coder carried out the main analysis and re-analyzed a randomly selected 10% of the posts for an intra-coder reliability test, which all indicated a good quality of the category system (Cohen’s Kappa > 0.8). Regarding the research question on the effectiveness of the communication strategy to facilitate user engagement and peer support frequency of and response to certain posting types was calculated. For this, after coding, the Excel file was imported to SPSS for descriptive data analysis. The average number of responses (including replies, reactions, and reply-reactions to posts) was calculated to analyze which types of posts facilitate user engagement: the mean was calculated by dividing the number of responses by the amount of postings of the type in question, e.g. if 50 postings were identified on topic A and 100 types of reactions to these postings were counted, a mean 2 was calculated indicating an average number of responses. A frequency analysis was conducted to analyze which types of peer support and topics were posted most frequently.

#### 2.3.4 Triangulation

For the results section in this article, the perception of the intervention was to be presented as comprehensively and with as many perspectives as possible. In order to meet this objective and with the aim of strengthening the credibility and confirmability of the results through methodological and data triangulation, the results of the different data collection approaches (supervision meetings with moderators, brief and in-depth interviews with participants and chat transcripts) were finally consolidated and structured in close discussion with the study team.

## 3 Results

The main findings are displayed in the following section, describing firstly the working procedure and role of moderators, secondly general challenges when realizing peer support via IMS that were observed in the intervention, and thirdly how the developed communication strategy to facilitate user engagement and peer support was perceived and showed effects in participants’ responses.

### 3.1 Moderating the IMS groups

#### 3.1.1 Moderators’ acceptance and use of manual

In the supervision meetings, all moderators indicated that they understood the manual well and were able to effectively work with it. Technical difficulties in sharing links and images occasionally arose but were resolved.

According to the moderators, the moderation task was rather time-consuming, and they felt the need to structure the workload and the working hours to protect themselves from being constantly involved in the task. Moderators reported spending approximately 6 and up to 12 hours per week on the moderation task, mainly for.

1. selecting the content of the manual, as they were instructed not to cover all contents of the extensive manual but rather to select from suggested materials. To post authentically, linked with personal experiences, moderators needed to prepare by familiarizing themselves with the content.
2. sharing the content: As the Mattermost App is operable also as desktop version, all moderators preferred to work via PC, instead of on their smartphones, as this made it more convenient to share content directly from the manual. This, however, led to the sharing of very long text passages that were less readable and appeared overwhelming on the participants’ smartphones. Furthermore, moderators – with their groups’ approval – scheduled times for posting content, e.g., twice a week, which resulting in multiple contents being posted at once and delayed responses. The analysis of chat protocols shows an average delay of 7h and 27min between posts and replies.

#### 3.1.2 Perception of the moderator’s role – self- and external (participants’) perspective

Moderators described their role as “equal team members” who, according to one moderator, possess a “slight knowledge advantage” due to the manual. They did not wish to dictate to other group members what is right or wrong but felt comfortable to share the content of the manual along with their personal experiences. All moderators seemed to struggle with their leading position concerning their responsibility towards the decreasing participation of group members or dealing with comments by group members that they perceived as critical. The supervision meetings with the moderators were therefore crucial for strengthening and motivating the moderators in their position and strategies.

Nearly all interview partners (IPs) described their perception of the moderator, when asked about their experience of the intervention. Thus, the role of the moderator as group leader was perceived as essential for the atmosphere and behavior within the group. The participants’ positive feelings towards the moderator appears to be fundamentally important:

“I believe that (…) is simply a wonderful person” (IP28, Pos. 36).

Furthermore, perceived efforts on the part of the moderator regarding the content were appreciated:

“I have to say, the moderator did a great job (…) was very dedicated and provided us with information very well.” (IP101, Pos. 15).

Simultaneously, dissatisfaction arose when there was a feeling that the moderator was not making an effort or did not provide a clear overview. A mistake by the moderator who inadvertently sent the same post twice – without realizing it – reinforced one IP’s (14) doubts about the moderator’s efforts. Even though the IPs credited the moderators for handling a challenging task, namely encouraging unknown individuals to engage to become a group, they criticized that the moderator did not respond empathetically enough to what was happening in the group, i.e., responded too slowly or not at all to posts of group members and, therefore, failed to initiate or sustain active “conversations” within the group.

In contrast, one IP (144) praised the same moderator for presenting content in a neutral and versatile manner (without always expressing an opinion). Neutrality and a certain degree of objectivity and group leadership seem to be desired not only concerning content but also in interactions with group members. Thus, dissatisfaction also arose when it was perceived that the moderator did not have equal relationships with all group members or engaged in additional private conversations or meetings with only a few group members (IP14).

### 3.2 Challenges to facilitate peer support in the IMS intervention

General challenges to facilitate peer support via IMS were observed and deduced from interview statements.

#### 3.2.1 Low group participation

The study team as well as the moderators and participants observed that the engagement of group members was initially high but decreased over time, with only a small number of participants posting regularly in the group. For the DiabPeerS study, randomization into intervention and control groups was based on coincidence rather than motivation to participate in the intervention. This led to the inclusion of individuals in the intervention group who were primarily interested in the assessment appointments rather than group interaction, as exemplified by one participant (IP15) who used their phone solely for important calls. Therefore, not all participants were interested in or accustomed to IMS communication. The incomplete and decreasing engagement of group members was perceived as concerning by all moderators, as it appeared to have a demotivating effect on the entire group:

“There were about 15 people in the group, and at the beginning, everyone participated. ‘I am so-and-so from here and there,’ but that quickly faded away. Only a few continued to actively participate, to write along. One participant said, ‘I read everything anyway, I just don’t write.’ In this case, this is just not really productive.” (IP14, Pos. 5).

Participants who did not engage in the interaction at all from the beginning were contacted by the study team to inquire about technical difficulties and received assistance in case of problems. However, technical difficulties seemed to account for only a minor part the decreasing engagement.

Results of the brief interviews show the reasons, listed by the participants, that prevented them from posting in the group (**Table 5**) and also general aspects that they felt were missing from the intervention (**Table 6**), which could also have reduced their motivation to engage:

**Table 5:** Summary: What prevents participants from posting in the group (brief interviews T1).

What prevents participants from posting in the group	Coding frequency
*No sense of participation (group anonymity)*	1
*No understanding, connection to online communication*	1
*Only interested in reading, observing*	7
*Feeling of having nothing relevant to say*	1
*No interest in personal exchange but interested in provided information*	1
*Technical difficulties (in several cases when technical problems were resolved, participation did not increase)*	5
*Unable to assess other group participants (group anonymity)*	1
*Moderator has a plan and program and determines what is posted (not participant)*	1
*No time, too much stress*	2

Explanation: Column 1: Summarized coded statements of participants (n=24) in brief interviews 3 months after start of intervention on factors that prevented them from posting in the group. Column 2: Frequency of codes (= how often statements were labeled with the code).

**Table 6:** Summary: What could be improved, or what was missing from the intervention, incl. classification reflecting type of peer support (brief interviews T1).

What was missing	Types of peer support	Coding frequency
*Desire for implementation (behavior change) assistance*	= need for DSME, not DSMS	2
*Desire for training (DSME)*	= need for DSME, not DSMS	1
*More participation of all group members*	= Negative user behavior hinders peer support	2
*Desire for mutual acquaintance/meeting*	= Anonymity of group hinders peer support	4
*Desire for more mutual motivation*	= Not sufficient emotional peer support	2
*Desire for clearer structure (including shorter texts)*	= Informational peer support needs structure	5

Explanation: Column 1: Summarized coded statements of participants (n=24) in brief interviews 3 months after start of intervention on factors that were missing or should be improved. Column 2: categorization to type or of peer support (informational, emotional, technical, medical, or sharing of experiences) Column 3: Frequency of codes (= how often statements were labeled with the code).

In the in-depth interviews, the incomplete participation of group members was also predominantly cited as an obstacle to peer support, and some participants and IPs explained that “*social media”* communication is simply “*not their thing*”, which is why they preferred to read/observe rather than post. Thus, the IMS group was not generally perceived as social media but described as a space where relevant content is shared, as opposed to online forums:

“For me, it’s not about the phone itself. I find WhatsApp groups to be fantastic (…) But it’s limited to meaningful things (…) But Instagram - which I used to have - in my opinion, out of 100 posts, 99 are unnecessary.” (IP28, Pos. 25-26).

#### 3.2.2 Group anonymity

In connection with the incomplete group participation and not being accustomed to IMS communication, the anonymity of the other group members also appeared to be barrier to group engagement. Some participants met each other during their assessment appointments, but the majority of group members did not know each other. On the one hand, this anonymity made it difficult for participants to interpret information shared by others, in the sense of whether the sharing person was particularly lazy/hardworking/sporty, etc. On the other hand, participants said they felt insecure about posting actively in the group because they experienced difficulties in assessing how their posts would be received:

“Often, you don’t know when you write about yourself (…) you don’t know, have I offended him or something. Because we don’t really know each other, you don’t know if the joke comes across the way it’s supposed to. So, the act of writing itself is not easy.” (IP101, Pos. 41).

Only one IP viewed the anonymity of the group as an advantage because it made them feel less restricted or obligated:

“From my point of view, it doesn’t matter whether I’ve met the person before. On the contrary: Maybe, if you don’t know each other, the threshold to write certain things or not write them is better. If I don’t have a personal connection to the people, I might find it easier to write certain things.” (IP28, Pos. 34).

To counteract anonymity and decreasing engagement, most IPs (also in the short interviews) suggested arranging regular meetings with interested parties or arranging at least one face-to-face meeting before the beginning of the intervention. Regular meetings to exercise together were mentioned particularly frequently in the interviews because physical activity holds significance in the IPs’ DSM, and exercising together is seen as more joyful than doing it alone (e.g., IP144, IP16, IP14). Additionally, it could help to break down barriers to physical activity, if it took place with individuals who also struggle with health issues and therefore supposedly practice sports at a similar level (IP46).

#### 3.2.3 Flexibility in terms of time and location

The IMS Intervention gave participants the opportunity to engage in an intervention aiming to benefit their DSM without time or location constraints, which has advantages but, seemingly, also disadvantages for group engagement.

Due to their life circumstances, IPs appreciated the flexible setting and found it accommodating and suitable for their needs. Even though some IPs would rather have participated in a more intense rehabilitation program, their circumstances would not allow it (IP46, IP101):

“As long as I’m working, I can’t just be available at any time of day or night … I’m very restricted. From that perspective, the method with the phone is very positive for me. I don’t have to go anywhere. I can do it at home.” (IP101, Pos. 67).

Being able to “*do it at home*” is connected with the opportunity to access information at one’s own pace and according to personal interests:

“What I really like about this group is that you can go back. If you say, there was something there, there was a topic. Then I can find it through the search function or I really scroll through again. I can still extract something with the links to the various posts.” (IP28, Pos. 25).

On the negative side, however, the lack of time and location constraints seems to lead to less commitment. Unlike in a group meeting, participants can easily delay reading and responding to messages or even neglect them altogether. Occasionally occurring technical issues with the messenger contributed to this negative user behavior. According to the participants, work and other obligations led them to contribute more or less to the group at different times:

“You don’t always have time, I work and then there was also a long time when I didn’t look at it.” (IP101, Pos. 15).

This “running in the background” or “on the side” of the intervention seems to lead to a perception of the intervention as an external regulating factor (in combination with regular assessment appointments): Individuals appreciate receiving “reminders” that prompt them to engage in activities related to their DSM or optimize it. Even participants who perceive themselves as successful in their DSM do not always find it easy to consistently apply all health-promoting behaviors and are grateful for regular reinforcement:

“I am someone who is very structured and takes care of his well-being, but often it’s just everyday life. Some things are unavoidable, which you wouldn’t normally do.” (IP144, Pos. 16).

Receiving reminders – which may cause guilt – is not perceived as joyful, but the advantage lies in feeling validated in existing health behaviors, being reminded of neglected aspects, and being informed about less familiar aspects. It can be assumed that this external regulation or lack of commitment diminishes the intensity of the intervention. However, whether the intervention was perceived as a personal benefit or not did not necessarily seem to depend on this, but rather on how the shared content was used, i.e., the readiness and ability to adapt information to one’s own purposes (see also 4.2).

### 3.3 Facilitating user engagement

#### 3.3.1 Response to nudging and call-to-action approaches

Regarding the use of nudging and call-to-action approaches to increase user engagement, moderators reported that playful approaches such as quizzes, as well as the sharing of experiences, elicited the most reactions. This is also confirmed by the results of the chat protocol analysis: Posts incorporating playful approaches, reminders (of goals or previously asked questions, etc.), mood questions, images, and reports on personal experiences generated more responses from participants than posts lacking these elements (see **Table 7**). In terms of recommending prompt responses to posts, there was an average delay of 7.27 hours between posting and reply/reaction. Although there are no specific time constraints regarding prompt responses to posts, this timeframe appears relatively lengthy.

**Table 7:** Responses to nudging and call-to-action approaches.

Posts labeled as different types of nudging/call to action	Mean of the number of responses	
	Including corresponding types of nudging and call-to-action approaches	Not including corresponding types of nudging and call-to-action approaches
**Playful approaches**	1.10 (2.194)	0.5 9(1.185)
**Reminders**	2.40 (2.444)	0.60 (1.228)
**Mood questions**	1.51 (1.954)	0.51 (1.096)
**Images**	0.72 (1.760)	0.61 (1.210)
**Reports on personal experiences**	0.74 (1.083)	0.60 (1.285)

Explanation: Column 1: Responses to posts labeled as nudging or call to action posts (structured into Playful approaches, Reminders, Mood questions, Images, Experience). Column 2/3: shows the mean of responses to posts with/without the identified nudging or to action approaches. The mean was calculated dividing the number of selected posts by the number of responses to this post. A number below 1 signifies, that there were more posts than responses.

Motivations to reply to posts were mentioned in the brief interviews by participants who did not post actively (see **Table 8**).

**Table 8:** Summary: motivation to post passively (brief interviews T1).

Motivation to passively post (respond) in the group		
*Politeness, feeling obliged to the moderator*		1
*Responding to questions*		3
*Avoiding black and white thinking, relativizing rules (when feeling a topic is being treated too one-sidedly)*	= informative/emotional support	1
*Participation in quizzes, interactive parts*		1

Explanation: Column 1: Summarized coded statements of participants (n=24) in brief interviews 3 months after start of intervention on factors that motivated them to post passively in the group. Column 2: categorization to type or of peer support (informational, emotional, technical, medical, or sharing of experiences) Column 3: Frequency of codes (= how often statements were labeled with the code).

A feeling of obligation towards the moderator in answering direct prompts and questions helped hesitant participants to overcome insecurities in posting. Nevertheless, a tendency to wait for someone else to respond first was also described:

“The question was sometimes unclear, so someone would respond, and I would be like “Ah, this is how it works.” Then you can respond, and it becomes easier. (IP46, Pos. 52).

Regarding the use of BCTs suggested in the manual, some difficulties were observed which could also be conceptual in nature: While the BCTs of Goal-Setting and Action Planning activities were carried out in all groups, they were not conducted in the originally planned format of SMART Goal-Setting and Action Planning a but in a reduced or modified form. For SMART Goal-Setting group members were provided with a guiding image on how to formulate a SMART goal (Specific, Measurable, Adequate, Relevant, Timely) by the moderators. However, group member did not seem to respond to this guidance and also no group conducted a joint evaluation or Problem-Solving activity as outlined in the manual. These activities seem to require a highly structured approach and more constant participation from both participants and moderators, which was not consistently present. However, these techniques were sometimes used spontaneously: One moderator created challenges such as “No sweets for the next four weeks” and invited group members to join, which was well-received. In other cases, group members posted health problems unrelated to formulated goals and sought advice from others. The analysis of chat protocols revealed that Goal Achievements (1.7% of all posts) as well as Behavior Changes (2.4% of all posts) were also posted without being linked to previously formulated goals within the group. However, 230 (8.9%) posts were labeled as Goal-Setting and showed to prompt more responses (mean = 0.75) than posts without Goal-Setting (mean = 0.61).

The results indicate that BCTs were applied in a spontaneous manner, and participants either applied BCTs on their own or upon invitation, reporting on intentions (Goal-Setting), results (Goal-Achievement), or difficulties (Problem-Solving). Generally, BCTs seem to be appreciated by participants as indicated by facilitating response, but the structured procedure in the manual does not seem to be applicable to the more flexible IMS setting.

#### 3.3.2 Response to types of peer support

Of all the posts, 55% were classified as peer support posts and further categorized into informational, emotional, technical, and medical peer support (see **Table 9**). In most categories, the majority of posts were created by moderators (see **Table 9**). How frequently participants responded to these types of posts is displayed in **Table 10**.

**Table 9:** Characteristics of peer support posts.

Type of peer support	Number of posts	% compared to peer-support posts (N = 1,415)	% compared to all posts (N = 2,574)	% posted by moderators
Informative support	1,072	75.8	41.6	56.1
Emotional support	493	34.8	19.2	61.9
Technical support	5	0.4	0.2	60.0
Medical support	3	0.2	0.1	0.0

Explanation: numbers of posts labelled as one type of peer support in % and proportion of peer support posts, posted by moderators in %.

**Table 10:** Responses to types of peer support.

Type of peer support	Mean of the number of responses	
	Including peer support	Not including peer support
Informative support	0.54*** (1.033)	0.68 (1.402)
Emotional support	0.69 (1.352)	0.60 (1.241)
Technical support	0.00 (0.000)	0.62 (1.264)
Medical support	0.67 (0.577)	0.62 (1.264)

Explanation: Column 1: Types of Peer Support. Column 2/3: shows the mean of responses to posts including/not including types of peer support. The mean was calculated dividing the number of selected posts by the number of responses to this post. A number below 1 signifies, that there were more posts than responses. Significant levels are indicated by asterisks, where * denotes p < 0.1, ** denotes p < 0.05, and *** denotes p < 0.01.

##### 3.3.2.1 Informational support

As indicated in **Table 9** the majority of peer support posts were classified as informational peer support (76%) and were created by moderators, which is in line with the task description of the moderators. Posts without informational support generated more engagement (response) than posts with informational support (see **Table 10**). This result requires further differentiation with regard to the topic on which information was shared.

###### 3.3.2.1.1 Response to topics of posts

A differentiation of informative content according to topics of the manual shows the quantity of posts shared per topic (N), and which topics provoked most responses (mean) (see **Table 11**). The majority of posts centered around nutrition, which correlates with the extensive content provided in the manual for this topic over an 8-week period, but the participants’ response was relatively low. Interestingly, posts related to monitoring and medication elicited higher participant engagement, despite their fewer numbers.

**Table 11:** Quantity of posts shared per topic and provoked responses.

Topic	N	%	Mean of responses
Explanation of DiabPeerS	409	15.9	0.69
Getting to know each other	144	5.6	0.67
Basics about DM2	143	5.6	0.73
Physical activity	289	11.2	0.72
Nutrition	639	24.8	0.51
Monitoring	365	14.2	0.73
Medication	60	2.3	0.82
Healthy coping	63	2.4	0.22
Other	462	17.9	0.54
Total	2574	100.0	0.62

Explanation: Posts per topic of the DiabPeerS manual in numbers and % as well as the mean of responses. The mean was calculated dividing the number of selected posts by the number of responses to this post. A number below 1 signifies, that there were more posts than responses.

On the one hand, this can be partly attributed to the operationalization of the analysis as – especially for large topics – a lot of content about a certain topic was frequently posted at once, and participants mostly replied to only part of this content, which led to a lower mean of responses in the calculation. However, posting a lot of content at once can have an especially overwhelming effect on participants causing fatigue in response.

While the amount of informational content was generally not described negatively in the interviews, it was noted that sometimes a lot of content was posted at once, and IPs described informative content as *“learning material”* that needed to be “*worked through*” (IP101), which they did not always manage. In combination with the results of the chat protocols, this suggests that participants faced information overload at times, and that the intervention was, to a certain extent, perceived as an educational program where content is shared by one responsible person, rather than a support program relying on the interaction of all.

Additionally – as stated by one IP 16: *“But these are actually always the things you get told.” (IP16, Pos. 55)* – the informative content was seemingly not new to all participants, which led to frustration and decreased engagement by some, while others viewed this positively as a *“good reminder”* and reinforcement in their individual DSM (IP101, IP144, IP28). The statement of IP16 indicates not only that the content was not new, but also a certain level of frustration with the amount of fairly general information – not tailored to the individuals’ needs – that PWDs are confronted with in the course of their treatment.

However, results suggest that two aspects seem relevant concerning the frustration of participants dealing with informative content: First, the combination of informative content with personal experiences (see 3.3.2.1.2) and second, the capability to extract personally relevant content from the shared materials and apply it effectively to one’s own situation (see 4 Discussion).

###### 3.3.2.1.2 Combining information with personal experiences

According to the IPs, it makes informative content more tangible if it is combined with experiential knowledge:

“Of course, I read many brochures and articles, but nonetheless, for me, it’s not as tangible as when someone else writes about it. When someone writes: I’m going for a run, and my blood sugar has dropped as a result. That’s more tangible for me because it’s shared by a tangible person, rather than reading it in some post.” (IP28, Pos. 44).

This is also supported by the analysis of chat protocols, indicating that posts containing personal experiences – being considered a nudging/call to-action approach – provoke a higher response (mean = 0.74) than post without them (mean = 0.60) (see **Table 7**).

In line with literature on peer support interventions for T2DM, it was important for the IPs to discuss diabetes topics with other affected individuals rather than health professionals or friends/family because a different level of understanding is provided (IP28). Most IPs perceived exchanging experiences with other PWDs as the primary benefit of the intervention – although this benefit was diminished due to the reduced participation (IP14, IP144, IP28, IP101) – and stated that reports on both negative and positive experiences were desired because it is not just about learning what works well but also about understanding what does not work for certain reasons (IP144). Sharing their own positive experiences can also motivate IPs to actively post in the group, not to be ostentatious but to encourage others:

“I think about physical activity, I voluntarily posted in the group. I have personally experienced how good I feel after exercising.” (IP144, Pos. 47).

The results of the brief interviews indicate that informative content was appreciated in the intervention, but beyond that, or in combination with the exchange of experiences, it was perceived as a benefit (see **Table 12**). Another important aspect, namely the opportunity to identify information of interest oneself, was also mentioned, suggesting that certain individuals possess strategies or skills to filter out relevant information for themselves, even amidst information overload. The appreciation of informative content seems to be connected with how the information is used and thus influences behavior, which became apparent when IPs described how they dealt with the information they received in the intervention.

**Table 12:** Summary: perceived benefit of participation in intervention (brief interviews T1).

Perceived benefit	Type of peer support	Coding frequency
*Good review, lots of information*	= Informative support	5
*Learn about others' strategies*	= Informative support / exchange of experiences	3
*Exchange of experiences*	= Exchange of experiences	4
*Opportunity to receive information of interest for oneself*	= Informative support	4
*Joint goal-setting as motivation*	= Use of behavioral change techniques	1

Explanation: Column 1: Summarized coded statements of participants (n=24) in brief interviews 3 months after start of intervention on perceived benefits from participating in the intervention. Column 2: categorization to type or of peer support (informational, emotional, technical, medical, or sharing of experiences) Column 3: Frequency of codes (= how often statements were labeled with the code).

###### 3.3.2.1.3 Identifying relevant information to change behavior

Two IPs, who perceived their participation in the intervention as beneficial, made statements about aspects contributing to successful behavior change. One IP described how they effectively applied a recommendation suggested by another group member on a certain topic, adapting it to a distinct context of personal relevance:

“The suggestion came from (…), which I then tried to implement further. I found that really great. (…) put it in there because it was about losing weight. For me, I don’t think I need to lose much more weight; for me, it was really about the sugar. I found that it works.” (IP144, Pos. 50).

This strategy for behavior change is grounded in the understanding that program suggestions and ideas require self-planned implementation for behavior change, which was verbalized by another IP:

“You receive a lot of information and everything. But then you have to do something yourself. But this is not only true for diabetes, but for many things. I mean, even if I attend various trainings – I still have to do it in the end. That’s why I think it’s great as it is.” (IP28, Pos. 50).

Past experiences with behavior changes that were carried out successfully were described as conducive to identifying and applying helpful measures/information from the shared content:

“Whereas such things come relatively easily to me due to my history. I had a heart attack and stroke, so my motivation is certainly greater than many others.” (IP28, Pos. 21).

In summary, the results on informational peer support suggest that an overload of informative content occurred, but the combination of informative content and personal experience made this content more tangible for participants. Furthermore, capabilities to identify relevant information and motivation to adapting and applying this information in the personal context facilitated behavior change and overall satisfaction with the intervention.

##### 3.3.2.2 Emotional support

The second-highest number of peer support posts (34.8%) were labeled as emotional support (see **Table 9**). These were posts that demonstrated understanding, provided positive feedback, expressions of care, encouragement, motivation, or validation of one’s experiences (others feel similarly). Contrary to informational support, posts labeled as emotional support prompted higher responses than posts without emotional support (see **Table 10**).

In the brief interviews, most motivations to post actively in the group were interpreted as emotional peer support (see **Table 13**), which indicates that providing and receiving emotional peer support generally facilitates group engagement.

**Table 13:** Summary: motivation to post in the group (brief interviews T1).

Motivation to post in the group	Type of peer support	Coding frequency
Motivation to actively post in the group		
*Wish to communicate, exchange*	= Informative/emotional support	1
*Share funny things*	= Emotional support	1
*Provide impulses, support*	= Emotional support	1
*Share experiences,knowledge*	= Sharing experiences / informative support	2
*Motivate others*	= Emotional support	1
*Share personal achievements (to motivate others)*	= Emotional support	2
*Reinforce positives, create a good atmosphere*	= Emotional support	

Explanation: Column 1: Summarized coded statements of participants (n=24) in brief interviews 3 months after start of intervention on factors that motivated them to post actively in the group. Column 2: categorization to type or of peer support (informational, emotional, technical, medical, or sharing of experiences) Column 3: Frequency of codes (= how often statements were labeled with the code).

However, it seems that emotional support was not provided equally in all groups and was not desired by all participants, as a number of controversial statements in the in-depth interviews suggest. Emotional support in the sense of mutual exchange and motivation for everyday challenges within DSM was described as missing by two IPs, but would have been desired:

“Things like ‘wow, how was your week? Or did you experience anything where you felt bad or had a setback?’ That never happened.” (IP14, Pos. 9).

At the same time, another IP from a different group complained about certain content being unnecessary that could in parts be identified as emotional support:

“There are some who seek for discussions in the group when they don’t have them at home (…) I don’t call anyone over video or have a video conference on ‘Yesterday I had so much sugar and today I have so much; and yesterday I ate too much and today too little.’ So, that’s not for me.” (IP127, Pos. 20-22).

Also, activities aiming at group cohesion and getting to know each other, such as sending vacation photos, were described by one IP as *“unnecessary”* group content (IP16). To put these negative statements of the latter two IPs into perspective, it needs to be explained that these IPs appeared to pursue predominantly specific content interests more related to medical support (see 3.3.2.3) and were not particularly interested in engaging in a PWD community.

##### 3.3.2.3 Medical and technical support

Only 0.4% and 0.2% of all peer support posts were labeled as technical and medical support, respectively (see **Table 9**). Posts labeled as technical or medical support prompted more response than posts without (see **Table 10**).

Technical support did occur only rarely, focusing only on specific aspects provided in the manual, i.e., blood glucose meter. Technical support may be more critical for individuals with insulin-dependent diabetes. During the measurement sessions, however, the use of continuous glucose monitoring devices was frequently discussed with the dietitian, but rarely mentioned in the group chats.

Medical support appears to be a “gray area” that overlaps with the realm of informative support. In the analysis of chat protocols, medical support would have only been identified if there were specific medication recommendations made. This did not occur in any case, and the moderators were instructed accordingly. However, medication was a topic mentioned in the manual for the purpose of sharing experiences and promoting medication adherence through information exchange, which is also recommended in the DSMES Standards (4).

In the in-depth interviews, two IPs expressed a very specific interest in experiences with medications and reduced or ceased their participation in the group when they did not receive corresponding responses:

“If someone had experience with (medication) for weight loss. But there was no experience, I have to gather it myself. With medical supervision.” (IP127, Pos. 28).

The other IP attempted to gather experiences with alternative treatment methods or the use of dietary supplements:

“I tried once to ask what other options there are. And I think I said once that I tried (alternative remedy), but nothing came back.” (IP16, Pos. 17).

Another observation related to medical support must be addressed: During the DiabPeerS intervention, some participants were prescribed GLP-1 analog medication as part of their treatment. This medication has gained media attention as a “weight loss injection” used by those who can afford it. However, frequent supply issues in Austria during the study period prevented some participants from accessing this medication despite having prescriptions. This situation may have limited discussions within the group, as some individuals may be reluctant to reveal that their improvements in blood sugar or weight were achieved through medication:

“I’m often not sure because even with (medication), you get the feeling you’re in an elite group. You almost feel guilty about outing yourself as taking (medication), because then it’s immediately like: Yeah, no wonder with (medication).” (IP101, Pos. 61).

In summary, results on medical support suggest that PWDs are interested in exchange about medication but seem hesitant to discuss medical aspects in the (lay) group and prefer consulting medical staff. For participants who are only interested in exchange about medical aspects, this limitation can become a barrier to group participation. However, difficulties in expressing specific interests in a way that can generate reactions within the group and making one’s voice heard may contribute to this barrier, which suggests that competencies in computer-mediated communication are required to enable a satisfactory participation experience.

## 4 Discussion

The article aimed to provide comprehensive information on the development and implementation of an intervention for peer support with the goal of enhancing DSMs via IMS, led by trained lay moderators. By analyzing qualitative data on participants’ and moderators’ perceptions as well as chat protocols, the study sought to identify factors contributing to the facilitation of peer support in an IMS setting. In this way, the study contributes to a differentiated perspective regarding the provision of different types of peer support in IMS interventions and delivers recommendations for the practical implementations of IMS-based diabetes self-management peer support interventions.

The development of the intervention was based on existing research findings on DSMES provided in peer support interventions. These findings were combined with insights from the field of DOCs and further refined using knowledge from the health communication science area of (online) user engagement. In the following section, the results of qualitative research approaches displayed above will be discussed focusing on the question of which factors of the intervention design facilitated or hindered peer support.

### 4.1 Diabetes self-management education or support?

Participants seem to have viewed the intervention as educational rather than supportive, which might have been caused by the structured manual and the moderator trained by health professionals but was not originally intended by the study team. The DSMES standards (4) integrate both education and support, which is expedient because both are interlinked in DSM guidance. For the discussion of the results, however, questions arise about [1] the inevitability of educational characteristics in DSM support, [2] the impact of an educational perception on user engagement, and [3] the characteristics and benefits of purely supportive interventions.

Ad [1] An evidence-based foundation provided by health professionals was needed for safety of the participants in the DiabPeerS intervention, to prevent the exchange of questionable views and avoid overburdening moderators, even if this empathized educational characteristics. However, this approach might have led to an overburdening of participants, especially when it comes to informational support (see 3.3.2.1), and might have diminished the empowering effect.

Ad [2] DSM education usually involves a health professional leading and instructing, while DSM support should emphasize the application of knowledge in daily life, highlight empowerment and participant expertise, which includes that participants (rather than health professionals) introduce topics or health issues to the discussion and take over an active role as peer supporter. It is questionable if the structured DiabPeerS intervention made participants adopt a passive role, reducing engagement as participants expressed doubts about the value of their contributions. Additionally, older participants in particular might be used to more traditional hierarchical relationships with health professionals and may not be accustomed to being active self-managers contributing to the therapy’s process with their expertise.

Ad [3] For future DSMS interventions, a methodological balance is needed that empathizes support, empowers participants, and prepares participants for appropriate roles and responsibilities. While DSMES standards offer an appropriate framework, there were concerns about repetitive general information, leading to frustration among PWDs. DSM support can mitigate this by providing tailored advice and a secure, engaging environment without commercial interests, by promoting the exchange of experiences among participants and encouraging participants to introduce the topics that they felt like discussing.

In this light, it becomes apparent that not only moderators but also participants of peer support interventions need preparational training to be able to fully engage in and benefit from a peer support intervention, which is why the following aspects should be considered for preparing participants.

### 4.2 Preparing participants to facilitate IMS engagement

Many participants described insecurities in posting and struggled with expressing themselves online. Research on Computer-Mediated Communication (CMC) competence shows that the ability to accurately and successfully communicate with others using computer-based technologies is essential for online group participation and discussion (61) and is affected by a person’s communication motivation, communication knowledge, communication skills, and contextual parameters (62). The results suggest that CMC competence was not distributed equally among the study population.

A study by Li, Zhang, and Ao (63) on user behavior of IMS applications shows that information overload in instant messaging software causes information anxiety, leading to delayed responses or none at all, which is considered negative user behavior. Environmental factors like task load and time pressure also contribute to negative user behavior. However, users are less likely to exhibit negative user behavior if they perceive the shared information as useful (63). These findings could also explain the behavior of participants in the DiabPeerS intervention: The large volume of informational content overwhelmed participants, thus leading to delayed or no responses. The non-binding nature of the IMS intervention, combined with individual time pressure and task load, further led to negative user behavior. However, negative user behavior can be mitigated if information is perceived as personally useful, but filtering personally useful information requires competencies: e-health literacy, defined as the capability to identify and define health issues, communicate, seek, understand, evaluate, and apply e-health information critically within cultural and social contexts to solve health problems (64), correlates positively with health-promoting and lifestyle behaviors (65).

Although not measured, interview statements suggest that participants who were capable to filter individually valuable information from the content engaged more and experienced the intervention as beneficial. This requires openness and flexibility towards program content. Those with rigid expectations were more likely to experience information overload and negative behavior, leading to a cycle of diminished participation and fewer meaningful exchanges. Like CMC competence, e-health literacy also seems to not have been distributed equally amongst participants.

### 4.3 Implications for practice

Facilitators for peer support lie in the program design and its theoretically sound embedding, providing an applicable framework and evidence-based content to engage PWDs in group interaction with the purpose of exchanging experiences on their daily DSM. To facilitate this exchange, however, implications for future implementations of the intervention program, based on the results of this study, should be considered.

#### 4.3.1 Training participants

To increase user engagement, a preparational face-to-face meeting prior to the intervention start is recommended to clarify the purpose of the intervention and discuss group members’ expectations concerning participation. Especially for persons who are not used to online or IMS communication, concerns that prevent individuals from posting in the group should be addressed. When it comes to persons who are generally not interested in communicating via IMS groups, participation in this type of intervention should be considered carefully.

When it comes to promoting user engagement to ensure that participants receive and contribute information extensively, the intervention should not aim to generally increase individuals’ media time, thereby contributing to digital burnout (66). The recommendations should primarily aim to increase the effectiveness of information usage and thus the perceived benefit, rather than simply extending screen time.

Although it may be a difficult task to increase e-health literacy in a single preparational meeting, participants should be made aware that it is their responsibility and decision how information shared in the program is used and applied. In addition, the reduction of informational content is recommended, which can make it easier to follow.

#### 4.3.2 Training moderators

The training focus for moderators should contain the overall explanation of the program, but an additional focus should be on guiding moderators in selecting and thereby reducing the content shared, especially when it comes to the content shared in one individual post. While reducing informational content, moderators should be encouraged to combine shared informational, emotional, technical, or medical content with prompts for discussion and/or interaction as well as invitations to share experiential knowledge.

Medical support is a sensitive issue in peer support interventions when provided by lay persons rather than health professionals. Recommendations for medication should be avoided in such settings. Medication is, however, a central aspect of disease self-management, and numerous unprofessional recommendations can be found online, especially for alternative medical products. An IMS intervention integrating support from health professionals, along with moderator training, supervision, and clear group guidelines appears to offer a protected space against product promotion and dubious unprofessional advice and thus provides an adequate framework for sharing experiences with medications as recommended in the DSMES standards (4), and PWDs should be encouraged to engage in exchange about their experiences instead of being afraid to address this topic.

## 5 Limitations

The aim of the article was to describe the development of an intervention in an RCT and provide additional information on how the intervention was perceived by the persons involved, in order to provide the reader with a complete picture of the intervention, in which not only the intention of the researchers but also the reception by the participants is presented. In this way, the content of the article can support researchers the design of peer-led IMS DSMES interventions. Results regarding the effects of the intervention will be published elsewhere.

As primarily qualitative research methods were employed to capture the perception of the intervention by participants, at this point a statement regarding the credibility, confirmability and transferability of the results is provided.

The prolonged engagement of the research team with the participants is a key aspect of this study. The majority of interviews were conducted by a single member of the study team, who was intimately involved in the intervention’s development and implementation. This individual observed all group chats, attended all measurement sessions, and participated in all training and supervision meetings with the moderators. The data analysis of the interviews and protocols was also conducted by the same individual, but where feasible, in collaboration with other members of the research team. (In particular, the coding was conducted by two members of the study team). This circumstance must be viewed with a degree of critical analysis and can be seen, to some extent, as both a limitation and a benefit. One potential drawback of the in-depth interviews is that socially desirable responses may have been provided, given the familiarity of the interviewer with the participants. However, particular care was taken during the sampling process to ensure an equal number of individuals who expressed satisfaction with the intervention and those who expressed dissatisfaction and had already done so. Thus, efforts were made to ensure that negative aspects were given sufficient attention. It should be noted that it was not the intention of the study team to present predominantly positive results; rather, the objective was to identify the barriers associated with such intervention designs. In this respect, there is a reduced risk of bias with regard to an overly positive portrayal of the perception of the intervention. However, there is an increased risk that the perspective of those people who are generally less extroverted when talking about their experiences is underrepresented. With regard to the sample size and the resulting data saturation, in the first step about 65% of the study population in the DiabPeerS intervention group were interviewed in the short interviews and thus a large part of the perspectives were recorded. In the in-depth interviews, some of these perspectives were repeated, but aspects were explained in much greater detail. As the interviewees were selected very carefully according to their diversity, it can be assumed that as many perspectives as possible were recorded in detail, thus achieving data saturation.

However, the strong involvement of the interviewer also has to be seen as an advantage, as the interviewer had a very precise understanding of what the interviewees meant when they reported their experiences and was able to link this to the intention of the developers and facilitators. This comprehensive perspective on the intervention was seen as an added value in the data analysis. Constant contact with the multi-professional study team throughout the intervention, and particularly during the discussion and analysis of the data, attempted to ensure reflexivity in dealing with personal conceptions. The triangulation of data carried out for this article allowed the researchers to corroborate the information gathered from different perspectives. Combining the more objective and quantitative analysis of chat transcripts with the interview results facilitated in-depth discussion of the results in the research team and increased the credibility of the interpretations by reducing the impact of potential biases.

Regarding the transferability of the findings, the authors provide a comprehensive description of the intervention setting and implementation, thus allowing readers to assess the applicability of the findings to similar situations: both the findings and the description of the development are transferable in the sense that they can be used to support other research projects.

Limitations were also observed in the analysis of chat protocols as writing styles in IMS are very diverse compared to other text forms e.g. regarding the use of short sentences, emojis, dialect expressions, etc. Evaluating the number of posts can thus be skewed regarding the frequency and amount of response post calculation, because some people split posts on one topic into several posts by hitting send in between, while others write longer posts. Also, the user interface of the IMS mattermost itself might have been a limitation to the acceptance and use of the intervention as most people are used to the functions of WhatsApp. Mattermost appears less user friendly and technical difficulties such as server outages did occur, at the same time mattermost – in contrast to many other IMS tools - provides comprehensive data security and the possibility to download IMS-chat protocols for scientific purposes.

## Data Availability

The datasets presented in this article are not readily available because of participants' confidentiality. Requests to access the datasets should be directed to ursula.hemetek@fhstp.ac.at.

## References

[1] World Health Organization . Herausgeber. In: Global report on diabetes, vol. 86. WHO Press, World Health Organization, Geneva (2016).

[2] Magliano D Boyko EJ . IDF diabetes atlas. 10th edition. Brussels: International Diabetes Federation (2021). 35914061

[3] Funnell MM Anderson RM . The problem with compliance in diabetes. JAMA. (2000) 284:1709. doi: 10.1001/jama.284.13.1709-JMS1004-6-1 11015809

[4] Beck J Greenwood DA Blanton L Bollinger ST Butcher MK Condon JE . 2017 National standards for diabetes self-management education and support. Diabetes Care. (2017) 40:1409–19. doi: 10.2337/dci17-0025 28754780

[5] American Diabetes Association . 5. Facilitating behavior change and well-being to improve health outcomes: standards of medical care in diabetes—2020. Diabetes Care. (2020) 43:S48–65. doi: 10.2337/dc20-s005"10.2337/dc20-S005 31862748

[6] International Diabetes Federation . Clinical Guidelines Task Force. Global guideline for type 2 diabetes. Brussels: International Diabetes Federation (2012).

[7] Österreichische Diabetes Gesellschaft . Diabetes mellitus-Anleitungen für die Praxis. Überarbeitete und erweiterte Fassung 2019. Wien Klin Wochenschr. (2019) 131:1–246. 10.1007/s00508-009-1263-y 19937307

[8] Paulweber B Valensi P Lindström J Lalic NM Greaves CJ McKee M . A european evidence-based guideline for the prevention of type 2 diabetes. Horm. Metab. Res. (2010) 42:S3–36. doi: 10.1055/s-0029-1240928 20391306

[9] Powers MA Bardsley J Cypress M Duker P Funnell MM Fischl AH . Diabetes self-management education and support in type 2 diabetes: A joint position statement of the american diabetes association, the american association of diabetes educators, and the academy of nutrition and dietetics. Clin. Diabetes Publ Am. Diabetes Assoc. (2016) 34:70–80. doi: 10.2337/diaclin.34.2.70 PMC4833481 27092016

[10] Davis J Fischl AH Beck J Browning L Carter A Condon JE . 2022 National standards for diabetes self-management education and support. Sci. Diabetes Self-Manag Care. (2022) 48:44–59. doi: 10.1177/26350106211072203 35049403

[11] Bekele BB Negash S Bogale B Tesfaye M Getachew D Weldekidan F . Effect of diabetes self-management education (DSME) on glycated hemoglobin (HbA1c) level among patients with T2DM: Systematic review and meta-analysis of randomized controlled trials. Diabetes Metab. Syndr. (2021) 15:177–85. doi: 10.1016/j.dsx.2020.12.030 33360516

[12] Odgers-Jewell K Ball LE Kelly JT Isenring EA Reidlinger DP Thomas R . Effectiveness of group-based self-management education for individuals with Type 2 diabetes: a systematic review with meta-analyses and meta-regression. Diabetes Med. J. Br. Diabetes Assoc. (2017) 34:1027–39. doi: 10.1111/dme.13340 28226200

[13] He X Li J Wang B Yao Q Li L Song R . Diabetes self-management education reduces risk of all-cause mortality in type 2 diabetes patients: a systematic review and meta-analysis. Endocrine. (2017) 55:712–31. doi: 10.1007/s12020-016-1168-2 27837440

[14] Minet L Møller S Vach W Wagner L Henriksen JE . Mediating the effect of self-care management intervention in type 2 diabetes: a meta-analysis of 47 randomised controlled trials. Patient Educ. Couns. (2010) 80:29–41. doi: 10.1016/j.pec.2009.09.033 19906503

[15] Steinsbekk A Rygg LØ Lisulo M Rise MB Fretheim A . Group based diabetes self-management education compared to routine treatment for people with type 2 diabetes mellitus. A systematic review with meta-analysis. BMC Health Serv. Res. (2012) 12:213. doi: 10.1186/1472-6963-12-213 22824531 PMC3418213

[16] Norris SL Lau J Smith SJ Schmid CH Engelgau MM . Self-management education for adults with type 2 diabetes: a meta-analysis of the effect on glycemic control. Diabetes Care. (2002) 25:1159–71. doi: 10.2337/diacare.25.7.1159 12087014

[17] Afshar R Tang TS Askari AS Sidhu R Brown H Sherifali D . Peer support interventions in type 2 diabetes: Review of components and process outcomes. J. Diabetes. (2020) 12:315–38. doi: 10.1111/1753-0407.12999 31639255

[18] Dennis CL . Peer support within a health care context: a concept analysis. Int. J. Nurs. Stud. (2003) 40:321–32. doi: 10.1016/s0020-7489(02)00092-5 12605954

[19] Dale JR Williams SM Bowyer V . What is the effect of peer support on diabetes outcomes in adults? A systematic review. Diabetes Med. (2012) 29:1361–77. doi: 10.1111/j.1464-5491.2012.03749.x 22804713

[20] Fisher EB Boothroyd RI Elstad EA Hays L Henes A Maslow GR . Peer support of complex health behaviors in prevention and disease management with special reference to diabetes: systematic reviews. Clin. Diabetes Endocrinol. (2017) 3:4. doi: 10.1186/s40842-017-0042-3 28702258 PMC5471959

[21] Liang D Jia R Zhou X Lu G Wu Z Yu J . The effectiveness of peer support on self-efficacy and self-management in people with type 2 diabetes: A meta-analysis. Patient Educ. Couns. (2021) 104:760–9. doi: 10.1016/j.pec.2020.11.011 33229189

[22] Heisler M . Overview of peer support models to improve diabetes self-management and clinical outcomes. Diabetes Spectr. (2007) 20:214–21. doi: 10.2337/diaspect.20.4.214

[23] Agastiya IMC Kurianto E Akalili H Wicaksana AL . The impact of telehealth on self-management of patients with type 2 diabetes: A systematic review on interventional studies. Diabetes Metab. Syndr. Clin. Res. Rev. (2022) 16:102485. doi: 10.1016/j.dsx.2022.102485 35512521

[24] Litchman ML Oser TK Hodgson L Heyman M Walker HR Deroze P . In-person and technology-mediated peer support in diabetes care: A systematic review of reviews and gap analysis. Diabetes Educ. (2020) 46:230–41. doi: 10.1177/0145721720913275 32321370

[25] Yang L Li K Liang Y Zhao Q Cui D Zhu X . Mediating role diet self-efficacy plays in the relationship between social support and diet self-management for patients with type 2 diabetes. Arch. Public Health. (2021) 79. https://www.ncbi.nlm.nih.gov/pmc/articles/PMC7849071/. 10.1186/s13690-021-00533-3 PMC7849071 33517902

[26] Gatlin TK Serafica R Johnson M . Systematic review of peer education intervention programmes among individuals with type 2 diabetes. J. Clin. Nurs. (2017) 26:4212–22. doi: 10.1111/jocn.13991 28793362

[27] Vorderstrasse A Lewinski A Melkus GD Johnson C . Social Support for Diabetes Self-Management via eHealth Interventions. Curr. Diabetes Rep. (2016) 16:56. doi: 10.1007/s11892-016-0756-0 27155606

[28] Tang TS Funnell M Sinco B Piatt G Palmisano G Spencer MS . Comparative effectiveness of peer leaders and community health workers in diabetes self-management support: results of a randomized controlled trial. Diabetes Care. (2014) 37:1525–34. doi: 10.2337/dc13-2161 PMC4030090 24722495

[29] Hilliard M Sparling K Hitchcock J Oser T Hood K . The emerging diabetes online community. Curr. Diabetes Rev. (2015) 11:261–72. doi: 10.2174/1573399811666150421123448 PMC4586085 25901500

[30] Laranjo L Arguel A Neves AL Gallagher AM Kaplan R Mortimer N . The influence of social networking sites on health behavior change: a systematic review and meta-analysis. J. Am. Med. Inform Assoc. JAMIA. (2015) 22:243–56. doi: 10.1136/amiajnl-2014-002841 PMC4433372 25005606

[31] Oser TK Oser SM Parascando JA Hessler-Jones D Sciamanna CN Sparling K . Social media in the diabetes community: a novel way to assess psychosocial needs in people with diabetes and their caregivers. Curr. Diabetes Rep. (2020) 20:10. doi: 10.1007/s11892-020-1294-3 32080765

[32] Statistik Austria . Erhebung über den IKT-Einsatz in Haushalten 2020. Vienna: IKT-Einsatz in Haushalten (2020). Available at: http://www.statistik.at/web_de/statistiken/energie_umwelt_innovation_mobilitaet/informationsgesellschaft/ikt-einsatz_in_haushalten/024571.html (Accessed April 09, 2021).

[33] Arnhold M Quade M Kirch W . Mobile applications for diabetics: a systematic review and expert-based usability evaluation considering the special requirements of diabetes patients age 50 years or older. J. Med. Internet Res. (2014) 16:e104. doi: 10.2196/jmir.2968 24718852 PMC4004144

[34] Conway N Campbell I Forbes P Cunningham S Wake D . mHealth applications for diabetes: User preference and implications for app development. Health Inf. J. (2016) 22:1111–20. doi: 10.1177/1460458215616265 26635324

[35] Gimbel R Shi L Williams JE Dye CJ Chen L Crawford P . Enhancing mHealth technology in the patient-centered medical home environment to activate patients with type 2 diabetes: A multisite feasibility study protocol. JMIR Res. Protoc. (2017) 6:e38. doi: 10.2196/resprot.6993 28264792 PMC5359418

[36] Kitsiou S Paré G Jaana M Gerber B . Effectiveness of mHealth interventions for patients with diabetes: An overview of systematic reviews. PloS One. (2017) 12:e0173160. doi: 10.1371/journal.pone.0173160 28249025 PMC5332111

[37] Wang X He L Zhu K Zhang S Xin L Xu W . An integrated model to evaluate the impact of social support on improving self-management of type 2 diabetes mellitus. BMC Med. Inform Decis Mak. (2019) 19. https://www.ncbi.nlm.nih.gov/pmc/articles/PMC6805520/. 10.1186/s12911-019-0914-9 PMC6805520 31640691

[38] World Health Organization . mHealth: new horizons for health through mobile technologies: second global survey on eHealth. Geneva: World Health Organization (2011). Available at: https://apps.who.int/iris/handle/10665/44607 (Accessed March 19, 2021).

[39] Tavakoli R Alipouran M Zarei F . Health belief model-based education through telegram instant messaging services on diabetic self-care. Health Educ. Health Promot. (2018) 6:91–6. doi: 10.29252/HEHP.6.3.91

[40] de Jongh T Gurol-Urganci I Vodopivec-Jamsek V Car J Atun R . Mobile phone messaging for facilitating self-management of long-term illnesses. Cochrane Database Syst. Rev. (2012) 12:CD007459. doi: 10.1002/14651858.CD007459.pub2 23235644 PMC6486189

[41] Tang PY Duni J Peeples MM Kowitt SD Bhushan NL Sokol RL . Complementarity of digital health and peer support: “This is what’s coming. Front. Clin. Diabetes Healthc. (2021) 2. doi: 10.3389/fcdhc.2021.646963 PMC10012094 36994335

[42] Simmons D Bunn C Cohn S Graffy J . What is the idea behind peer-to-peer support in diabetes? Diabetes Manag. Diabetes Management (2013) 3:61–70. doi: 10.2217/dmt.12.80

[43] Höld E Grüblbauer J Wiesholzer M Wewerka-Kreimel D Stieger S Kuschei W . Improving glycemic control in patients with type 2 diabetes mellitus through a peer support instant messaging service intervention (DiabPeerS): study protocol for a randomized controlled trial. Trials. (2022) 23:308. doi: 10.1186/s13063-022-06202-2 35422003 PMC9009500

[44] Anderson RM Funnell MM . Patient empowerment: reflections on the challenge of fostering the adoption of a new paradigm. Patient Educ. Couns. (2005) 57:153–7. doi: 10.1016/j.pec.2004.05.008 15911187

[45] Haslbeck JW Schaeffer D . Selbstmanagementförderung bei chronischer Krankheit: Geschichte, Konzept und Herausforderungen. Pflege. (2007) 20:82–92. doi: 10.1024/1012-5302.20.2.82 17658008

[46] Kolb L . An effective model of diabetes care and education: the ADCES7 self-care behaviors^TM^ . Sci. Diabetes Self-Manag Care. (2021) 47:30–53. doi: 10.1177/0145721720978154 34078208

[47] Mulcahy K Maryniuk M Peeples M Peyrot M Tomky D Weaver T . Diabetes self-management education core outcomes measures. Diabetes Educ. (2003) 29:768–70, 773–84, 787-788. doi: 10.1177/014572170302900509 14603868

[48] Peers for Progress . Peers for Progress Diabetes Peer Support Training (2014). Available online at: http://peersforprogress.org/wp-content/uploads/2014/01/20140102_diabetes_peer_supporter_training_curriculum.pdf (Accessed November 20, 2020).

[49] Tang TS Funnell M . Peer Leader Manual.Michigan: International Diabetes Federation (2017). Available at: https://www.idf.org/e-library/education/65-idf-peer-leader-training-manual.html (Accessed April 12, 2021).

[50] van Vugt M de Wit M Cleijne WH Snoek FJ . Use of behavioral change techniques in web-based self-management programs for type 2 diabetes patients: systematic review. J. Med. Internet Res. (2013) 15. https://www.ncbi.nlm.nih.gov/pmc/articles/PMC3869055/. 10.2196/jmir.2800 PMC3869055 24334230

[51] Gavrila V Garrity A Hirschfeld E Edwards B Lee JM . Peer support through a diabetes social media community. J. Diabetes Sci. Technol. (2019) 13:493–7. doi: 10.1177/1932296818818828 PMC6501537 30600704

[52] Litchman ML Rothwell E Edelman LS . The diabetes online community: Older adults supporting self-care through peer health. Patient Educ. Couns. (2018) 101:518–23. doi: 10.1016/j.pec.2017.08.023 28947360

[53] Grunberg PH Dennis CL Da Costa D Gagné K Idelson R Zelkowitz P . Development and evaluation of an online infertility peer supporter training program. Patient Educ. Couns. (2020) 103:1005–12. doi: 10.1016/j.pec.2019.11.019 31761526

[54] Pedersen EA Loft LH Jacobsen SU Søborg B Bigaard J . Strategic health communication on social media: Insights from a Danish social media campaign to address HPV vaccination hesitancy. Vaccine. (2020) 38:4909–15. doi: 10.1016/j.vaccine.2020.05.061 32482460

[55] Thaler RH Sunstein CR . Nudge: improving decisions about health, wealth, and happiness. New Haven: Yale University Press (2008). p. 293.

[56] Sunstein CR . Nudging: a very short guide. Bus Econ. (2019) 54:127–9. doi: 10.1057/s11369-018-00104-5

[57] Patel MS Benjamin EJ Volpp KG Fox CS Small DS Massaro JM . Effect of a game-based intervention designed to enhance social incentives to increase physical activity among families: the BE FIT randomized clinical trial. JAMA Intern. Med. (2017) 177:1586. doi: 10.1001/jamainternmed.2017.3458 28973115 PMC5710273

[58] Kwan YH Cheng TY Yoon S Ho LYC Huang CW Chew EH . A systematic review of nudge theories and strategies used to influence adult health behaviour and outcome in diabetes management. Diabetes Metab. (2020) 46:450–60. doi: 10.1016/j.diabet.2020.04.002 32387700

[59] Kuckartz U Rädiker S . Qualitative content analysis: methods, practice and software. 2nd edition. Los Angeles, London, New Delhi, Singapore, Washington DC, Melbourne: SAGE (2023). p. 236.

[60] Flick U . Qualitative Sozialforschung: eine Einführung. 10. Auflage, Originalausgabe. Reinbek bei Hamburg: rowohlts enzyklopädie im Rowohlt Taschenbuch Verlag. (Reinbek bei Hamburg: rowohlts enzyklopädie im Rowohlt Taschenbuch Verlag) (2021). p. 623

[61] Chih-Ming C Ying-You L . Developing a computer-mediated communication competence forecasting model based on learning behavior features. Comput. Educ. Artif. Intell. (2020) 1:100004. doi: 10.1016/j.caeai.2020.100004

[62] Spitzberg BH . Preliminary development of a model and measure of computer-mediated communication (CMC) competence. J. Comput-Mediat Commun. Januar. (2006) 11:629–66. doi: 10.1111/j.1083-6101.2006.00030.x

[63] Li J Zhang S Ao W . Why is instant messaging not instant? Understanding users’ negative use behavior of instant messaging software. Comput. Hum. Behav. (2023) 142:107655. doi: 10.1016/j.chb.2023.107655

[64] Gilstad H . Toward a comprehensive model of eHealth literacy. Proceedings of the 2nd European Workshop on Practical Aspects of Health Informatics(PAHI 2014), Trondheim Norway, (2014). doi: 10.13140/2.1.4569.0247.

[65] Kim K Shin S Kim S Lee E . The relation between eHealth literacy and health-related behaviors: systematic review and meta-analysis. J. Med. Internet Res. (2023) 25:e40778. doi: 10.2196/40778 36716080 PMC9926349

[66] Markowetz A . Digitaler Burnout: warum unsere permanente Smartphone-Nutzung gefährlich ist. München: Droemer (2015). p. 220.

